# *Psidium guajava* L. phenolic compound-reinforced lamellar scaffold for tracheal tissue engineering

**DOI:** 10.1007/s13346-023-01381-0

**Published:** 2023-08-11

**Authors:** Venâncio A. Amaral, Juliana Ferreira de Souza, Thais F. R. Alves, José M. de Oliveira Junior, Patrícia Severino, Norberto Aranha, Eliana B. Souto, Marco V. Chaud

**Affiliations:** 1 Laboratory of Biomaterials and Nanotechnology, University of Sorocaba, UNISO, Raposo Tavares, Sorocaba, São Paulo 18023-000 Brazil; 2 Laboratory of Applied Nuclear Physics, University of Sorocaba, UNISO, Raposo Tavares, Sorocaba, São Paulo 18023-000 Brazil; 3 Institute of Technology and Research, Tiradentes University, Murilo Dantas, Aracaju, Sergipe, 300 Brazil; 4 College of Engineering of Bioprocess and Biotechnology, University of Sorocaba, UNISO, Raposo Tavares, Sorocaba, 18023-000 Brazil; 5 Laboratory of Pharmaceutical Technology, Department of Drug Sciences, Faculty of Pharmacy, University of Porto, Jorge de Viterbo Ferreira, 4050-313 Porto, Portugal; 6 MEDTECH, Department of Drug Sciences, Faculty of Pharmacy, University of Porto, 4050-313 Porto, Portugal; 7 Associate Laboratory i4HB-Institute for Health and Bioeconomy, Faculty of Pharmacy, University of Porto, 4050-313 Porto, Portugal

**Keywords:** Trachea, Tracheal cancer, Total phenolic compounds, Dense lamellar scaffold, Plastic compression

## Abstract

The aim of this work was to develop a dense lamellar scaffold, as a biomimetic material with potential applications in the regeneration of tracheal tissue after surgical tumor resection. The scaffolds were produced by plastic compression technique, exploiting the use of total phenolic compounds (TPC) from *Psidium guajava* Linn as a potential cross-linking agent in a polymeric mixture based on collagen (COL), silk fibroin (SF), and polyethylene glycol 400 (PEG 400). Fourier transform infrared spectroscopy (FTIR) and differential scanning calorimetry (DSC) confirmed the chemical interactions between the polymers and the cross-linking of TPC between COL and SF. Morphological analyses showed scaffolds with porosity, interconnectivity, and a porous surface structure with a gyroid-like geometry. The analysis of the anisotropic degree resulted in anisotropic structures (0.1% TFC and 0.3% TFC) and an isotropic structure (0.5% TFC). In the mechanical properties, it was evidenced greater resistance for the 0.3% TFC formulation. The addition of TPC percentages did not result in a significant difference (*p* > 0.05) in swelling capacity and disintegration rate. The results confirmed that TPC were able to modulate the morphological, morphometric, and mechanical properties of scaffolds. Thus, this study describes a potential new material to improve the regeneration of major tracheal structures after surgical tumor removal.

**Graphical Abstract:**

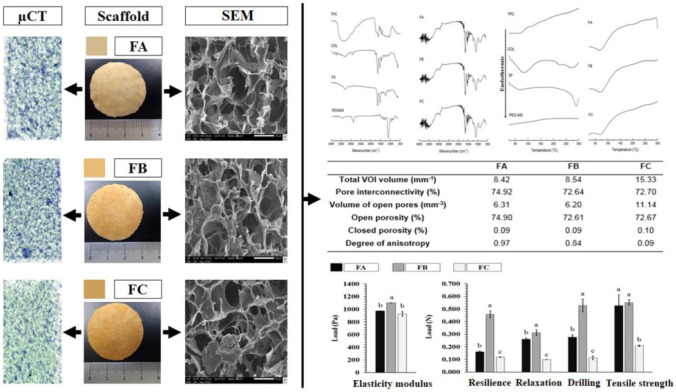

## Introduction

Trachea is a portion of the respiratory tract, performing multiple functions, such as conducting, filtering, heating, and humidifying the air for gas exchange from the external environment to the lungs [1]. This organ is a resistant but flexible hollow tubular structure, presenting a complex geometry of pseudostratified cylindrical ciliated epithelium, connective tissue, smooth muscle, and C-shaped cartilage rings [2, 3].

Airflow in the airways can be obstructed by diseases such as stenosis, cancer, congenital anomalies, or trauma [4]. The tumors are rare trachea-related diseases and represent a small proportion of respiratory tract neoplasms, comprising about 2% of airway neoplasms [5, 6]. However, tracheal cancer has a treatment option when the tumor is localized, even representing a low-incidence disease [7]. This option consists of complete tracheal resection with end-to-end anastomosis. This technic is the best treatment option for tracheal tumors when there is enough healthy tissue to allow juxtaposition, presenting better results in terms of overall survival [8].

Due to its rarity and associated symptoms that are often nonspecific, the diagnosis of patients with tracheal cancer can take from 2 months to 2 years. As a result, patients may have a disease that cannot be resected and, consequently, a reduction in the survival rate of patients [4]. The repair of significant tracheal defects remains a challenging surgical problem. Thus, alternative approaches for anatomical and functional rehabilitation of the trachea have been extensively investigated by tissue engineering and regenerative medicine [1, 9]. Thus, tracheal tissue engineering aims to promote the healing and regeneration of tracheal tissue structures to restore mechanical and physiological functions [10]. However, few alternative therapies are available for patients with severe tracheal diseases [11].

Scaffolds are three-dimensional structures used for tissue engineering, to restore, maintain, or improve tissues. These structures are instrumental in supporting the growth of cells, shaping the formation of functional tissues and organs as they guide cells to replicate and to synthesize extracellular matrix and other biological molecules. Scaffolds can be obtained from a diversity of synthetic and natural materials [12], e.g., linear aliphatic polyesters (polyglycolic acid, polylactic acid, polylactic acid-co-glycolic acid, poly-ε-caprolactone, polyhydroxy butyrate), polyphosphoesters, polyphosphazenes, polyethylene glycol, polyacrylic acid, polyvinyl alcohol, proteins (e.g., collagen, silk fibroin, fibronectin, laminin), polysaccharides (e.g., chitosan, alginate, hyaluronate), and inorganic materials (e.g., porous bioactive glasses and calcium phosphates used in bone and other mineralized tissue engineering) [13, 14]. The selection of the materials is governed by the type of tissue to engineer and respective functions, whether they should retain their structure in vitro or in vivo.

Collagen is a major component of the extracellular matrix commonly used in the repair of soft tissue. This fibrous protein has been combined with other polysaccharide macromolecules forming copolymers (e.g., collagen-glycosaminoglycan for heart valve structure engineering [15]) to exploit its biological properties. Its irreversible denaturated form (i.e. gelatine) is also being used as an alternative to collagen, in cell culture and tissue regeneration. Gelatine can be cross-linked with other polymers with the aim to create composites with improved mechanical properties and increased biocompatibility [16].

Silk fibroin is obtained from silkworm silk and is known for its tunable mechanical properties, biocompatibility, bioresorbability, biodegradability, and low immunogenicity [17]. Besides, silk fibroin is approved by the Food and Drug Administration (FDA) for biomedical applications [18]. The protein can be used in the engineering of cartilages, ligaments, tendons, skin, bones, and other membranes. Recently, it has also been used in the production of methacrylated photocurable silk fibroin (SF) bioink for three-dimensional (3D) printing [19].

Polyethylene glycol 400 is also an important hydrophilic compound for the production of hydrogels that can also be used in tissue engineering research [20]. PEGs offer a high swollen capacity to create 3D structures similar to soft tissues.

*Psidium guajava* Linn is a plant that belongs to the Myrtaceae family and is found in several tropical and sub-tropical regions [21]. Its use in folk medicine is widely known, provided that its biological activities are attributed to the high level of polyphenolic compounds [22].

The aim of this study was to develop dense lamellar scaffolds obtained by plastic compression technique, from hydrogels based on collagen, silk fibroin, polyethylene glycol 400. The total phenolic compounds (TPCs) extracted from guava leaves (*Psidium guajava* Linn.) were used as a crosslinking agent. The biomaterial was characterized with respect to its mechanical and morphological properties, swelling capacity, and in vitro biodisintegration rate. From our findings, this novel material can be exploited in the tissue engineering of tracheal structures, such as cartilage, smooth muscle, and connective tissue, to restore its mechanical and physiological functions. Currently, the options for regenerating the tracheal epithelium through tissue engineering are limited. However, earlier studies have comprehensively characterized biomaterials with the purpose of tracheal reconstruction [23]. COL and SF were selected with a critical focus on mimicking the extracellular cartilage matrix and following specific criteria of compatibility with the extracellular matrix for cell, early focal attachment spreading and differentiation, required mechanical properties, and bulk mass. PEGs offer high swollen capacity to create 3D structures similar to soft tissues by plastic compression technique. Although fibroin leads to delayed ciliated cell development, we used SF, COL, PEG, and TPC to develop a composite biomaterial that offers both biomaterial mechanical and cells differentiation advantages and meets all our selection criteria. SF possesses several impressive properties that make it suitable for tissue engineering, including oxygen and water permeability, low thrombogenicity, and minimal inflammatory response.

## Materials and methods

### Materials

NovaProm Food Ingredients Ltda (São Paulo, Brazil) supplied collagen type B powder extracted from bovine hides. Polyethylene glycol 400 was obtained from Dinâmica (São Paulo, Brazil). Silkworm *Bombyx mori* cocoons were used to extract silk fibroin. Total phenolic compounds were extracted from guava leaf (*Psidium guajava* Linn.), and ultrapure water (18.2 MΩ.cm ^−1^) was home supplied. The other reagents were pharmaceutical grade.

### Extraction of silk fibroin

The process of extracting the *Bombyx mori* silk fibroin was adapted from Komatsu et al. [24], as previously described by us [25]. Briefly, the silkworm cocoons were cut into small pieces, added to aqueous Na_2_CO_3_ solution (0.5 wt %), and kept at 120 °C for 15 min in an autoclave (80 Kps). In this process, sericin extraction occurs from the cocoons, and subsequently, the silk fibroin was washed entirely with purified water to extract the residues of the sericin and dried in an oven at 50 °C for 24 h. The degummed silk fibroin was dissolved in 1 L of a ternary solution of CaCl_2_.2H_2_O/CH_3_CH_2_OH/H_2_O (mole ratio 1:2:6) at 85 °C; this dispersion was filtered and dialyzed on a cellulose membrane with a molecular weight limit of 14,000 Da, for 3 days under magnetic stirring to produce the fibroin solution. The final concentration of fibroin was about 2 to 3% by weight, which was determined by weighing the remaining solid after drying.

### Extraction of total phenolic content (TPC)

The process of extracting the total phenolic compounds (TPC) was carried out as described by Amaral et al. [26]. The ultrasound-assisted extraction was carried out using hydroethanolic solvent 70% (v/v), acidified with hydrochloric acid (0.5% v/v), and applied ultrasonic wave single frequency (40 kHz). For this extraction, 10 g of standardized guava leaf powder was placed in Erlenmeyer, dispersed in 150 mL of previously acidified ethanol 70% v/v, and kept in an ultrasound bath (Unique, USC-3300, São Paulo, Brazil) for 60 min, at 30 °C. Both dispersions were vacuum filtered (Whatman® cotton filter, grade 44:3 μm) and stored away from light and heat. The residue retained in the cotton filter was reprocessed using the same extraction conditions described. The product from the first and second extraction processes was mixed. The solvent was eliminated using a rotatory evaporator (Tecnal, TE-211, São Paulo, Brazil). The concentrated extract was resuspended in ultrapure water and dried by lyophilization (Lyofilizer-L101, Liotop, São Paulo, Brazil). The dry extract of guava leaves from acidified ethanol (50%) and ethanol (70%) was stored away from humidity, light, and heat.

### Preparation of the COL-SF-PEG 400 hydrogel with the addition of TPC

The hydrogel formulation was prepared based on a dispersion of collagen (COL) in purified water. Silk fibroin (SF) and polyethylene glycol (PEG) 400 hydrodispersible polymers were dispersed in the aqueous solution of COL; then, percentages of TPC were added as described in Table 1. The polymer mixture was stirred until complete homogenization. Subsequently, the final pH of the formulation was adjusted to 10 using a 2 M NaOH solution. The obtained dispersion was stored in cylindrical plastic containers (internal diameter = 21 mm and height = 11 mm) and incubated at 10 ± 2 °C for 24 h for polymerization.

**Table 1:** Composition of formulations for the preparation of hydrogel-based scaffolds

Components (%, mass)	Formulations		
	**FA**	**FB**	**FC**
Collagen (COL)	5.00	5.00	5.00
Silk fibroin (SF)	1.00	1.00	1.00
Polyethylene glycol 400 (PEG 400)	1.25	1.25	1.25
Total phenolic compounds (TPC)	0.10	0.30	0.50
Ultrapure water q.s.p	92.65	92.45	92.25
Total	100.00	100.00	100.00

### Preparation of lamellar scaffolds using a plastic compression technique

The scaffolds were obtained using the plastic compression technique to produce three types of hydrogel formulations (i.e., FA, FB, and FC), using a hydraulic press (Shimadzu, SSP-10th. Kyoto, Japan) following the work by Alves et al. [27] with the freezing temperature at − 50 °C. Each hydrogel was placed between layers of nylon mesh (50 µm) and metallic mesh, applying compressive stress with a load of 4 kN applied for 10 min to remove water and to produce a dense biomaterial. After compression of the hydrogel, the obtained polymer matrix was frozen at − 50 °C (UFR30, Liotop, São Paulo, Brazil) for 24 h and, subsequently, lyophilized (Lyophilizer–L101, Liotop, São Paulo, Brazil).

### Fourier transform infrared spectroscopy

The Fourier transform infrared** (**FTIR) spectra were obtained on LabSolutions software IR s.v.2.10 (Shimadzu, IRAffinity-1, Kyoto, Japan). Prior to each analysis, each tested scaffold was carefully placed on the Attenuated total reflectance (ATR-8200HA) support, and analysis was done over the range between 4000 and 600 cm^−1^ at 4 cm^−1^ resolutions, to record the stretches of the chemical bonds between the main functional groups of each molecule present in the tested samples, making a total average of 128 scans.

### Determination of the zeta potential of total phenolic compounds

For the total phenolic compounds (TPC) zeta potential test (BrookHaven–NanoBrook-90 Plus, New York, USA), the equipment was adjusted to a wavelength of 635 nm and a fixed angle of 90°. The samples were prepared by solubilizing the TPC in ultrapure water, obtaining solutions at concentrations of 0.1, 0.2, and 0.5% (m/v). After solubilization, the pH of the samples was determined to obtain two groups (initial and final), and in the final group, the pH was adjusted to 10, using a 2 M NaOH solution. The assay was performed in an electrophoretic cell and analyzed at 25 °C in triplicate. The result of the thermodynamic stability of the zeta potential was expressed in millivolts (mV).

### Differential scanning calorimetry

For each tested sample (individual components, lyophilized scaffolds, and hydrated scaffolds), a mass of 2 mg was placed in an aluminum, which was then hermetically crimped and heated from 25 to 300 °C at a rate of 10 °C/min, under dry nitrogen purged at 50 mL/min, in a differential scanning calorimeter (Thermal Analyzer TA 60WS, DSC-60, Shimadzu, Kyoto, Japan).

### Scanning electron microscopy

For the scanning electron microscopy (SEM) analysis, samples were placed onto an aluminum sample holder and fixed using double-sided carbon adhesive tape. Samples were then surfaced with a thin gold layer per metallizer (DII-29010SCTR Smart Coater, JEOL, Tokyo, Japan), spraying for 4 min at 3 mA, and SEM analysis was run in a scanning electron microscope (JSM-IT200, JEOL, Tokyo, Japan) with an accelerating electrical voltage of 15 kV and a beam current of 100 pA.

### Computed microtomography

Computed microtomography (μCT) was used to analyze the morphometric properties of the pores and scaffold porosity (%), their size, interconnectivity, and degree of anisotropy. X-ray microtomography (Brucker-micro CT—SkyScan 1174, Kontich, Belgium) was used to record the scaffolds’ images, using a high-resolution scanner, with 28 mM pixel and 1.7 s integration time. A total of 790 mA of current and 34 keV of energy were implemented from the x-ray source, which was filtered through an Al filter. Projections were recorded in a 180° range with an angular step of 1° rotation, building up 3D virtual models that are representative of various regions of the scaffolds, using the CT Analyzer v.1.13.5 software for data curation and treatment.

### Mechanical properties

The mechanical properties (tensile strength, elasticity, drilling or perforation, relaxation, and resilience) of the scaffolds were analyzed in a texture analyzer (Stable Micro Systems-TA-XT Plus, Surrey, UK) using a load cell of 5 kg and a test velocity set for a rate of 2 mm/s for the drilling and tensile strength tests and for a rate of 0.5 mm/s for elastic modulus, resilience, and relaxation tests. Prior to the texture analysis, the scaffolds were firstly hydrated for 1 h in water and applied an area of 6 cm^2^. A force of 0.098 N was used for all tests (*n* = 3).

### Swelling efficiency

The swelling efficiency test was implemented following the procedure modified by Alves et al. [23]. Prior to the test, scaffolds were cut into a square format (1 cm^2^), weighed, and then immersed in 3 mL of phosphate-buffered saline (PBS) pH 7.4 at 37 ± 1 °C for up to 120 h.

At pre-determined times (24, 48, 72, 96, and 120 h), the scaffold was removed, and the ability to retain PBS liquid was measured. Firstly, the capacity to absorb PBS was determined for the scaffold structure as a whole; this means the material itself and the pore system. The samples were removed from the liquid at each time point, shaken gently, and weighed without dripping to determine the weighed without dripping (Wwd). The second measurement was done after pressing and “drying” the same scaffold embedded between the filter paper sheets to remove the PBS stuck in its porous structure (Wwp). The scaffold was then dried at 37 ± 1 °C until it reached constant weight (Wd), and the percentage of uptaken liquid was determined by applying the following equation [25]:

$$\mathrm{Fluid\ uptake\ of\ scaffold}\, ({\%})=\left(\frac{Ww-Wd}{Wd}\right) \times 100$$

where *Ww* represents either the weighed without dripping (Wwd) or the porous structure (Wwp) and *Wd* is the dried scaffold. Each sample was measured in triplicate.

### In vitro biodisintegration study

The in vitro biodisintegration test was implemented following the procedure modified by Alves et al. [23]. The blends were firstly hydrated in phosphate-buffered saline (PBS) pH 7.4 at 37 ± 1 °C to determine their degree of disintegration. The blends were then cut into a 1 cm^2^ square shape and weighed prior to the disintegration test to determine *Wd’*, and then immersed in 3 ml PBS at 37 ± 1 °C for 120 h. At pre-determined times (24, 48, 72, 96, and 120 h), blends were removed, washed using a large volume of deionized water to remove buffer salts, and dried at 37 ± 1 °C until reaching constant weight. Blends were finally weighted (Wa), and the weight loss percentage was calculated using the following equation [25]:

$$\mathrm{Weight\ loss}\, ({\%})=100 \times \left(\frac{{Wd}^{^{\prime}}-Wa}{{Wd}^{^{\prime}}}\right)$$

where *Wd’* and *Wa* are the weight recorded before and after the disintegration test, respectively. The pH value of PBS was recorded in triplicate for each time point using a pH meter (Tecnal, TE-5, Piracicaba, Brazil).

### Statistical analysis

The results are shown as the average of *n* = 3 and the respective standard deviation (S.D.). For the statistical analysis, ANOVA followed by the Tukey test was used, considering statistically significant the difference of results for *p* value < 0.05.

## Results and discussion

### Macroscopic characterization of the dense lamellar scaffold

Production techniques have a significant impact on the properties of porous scaffolds [14]. In this work, a plastic compression technique was used to obtain three distinct hydrogel-based scaffolds (Table 1). The plastic compression technique was selected to see if collagen could be used in the formulation since it plays a crucial role in mimicking the cellular matrix.

After the incubation period, the polymeric dispersions of COL, SF, and PEG 400 with variations of TPC (0.1, 0.3, and 0.5%) generated three-dimensional homogeneous hydrogels with excellent handling resistance. Then, the hydrogels were subjected to plastic compression and directed to freezing. The compression causes partial water removal. However, the residual water portion in the formulations plays an essential role in forming pores. The ice crystal’s growth pushes the polymers apart, and the matrix is restructured more adequately during the freezing process. With the lyophilization of the formulations, the ice crystals are eliminated by sublimation. This process provides the scaffolds with a unique porous architecture in which the degree of porosity is highly linked to the crystallography exerted by the solvent [28].

**Fig. 1:**
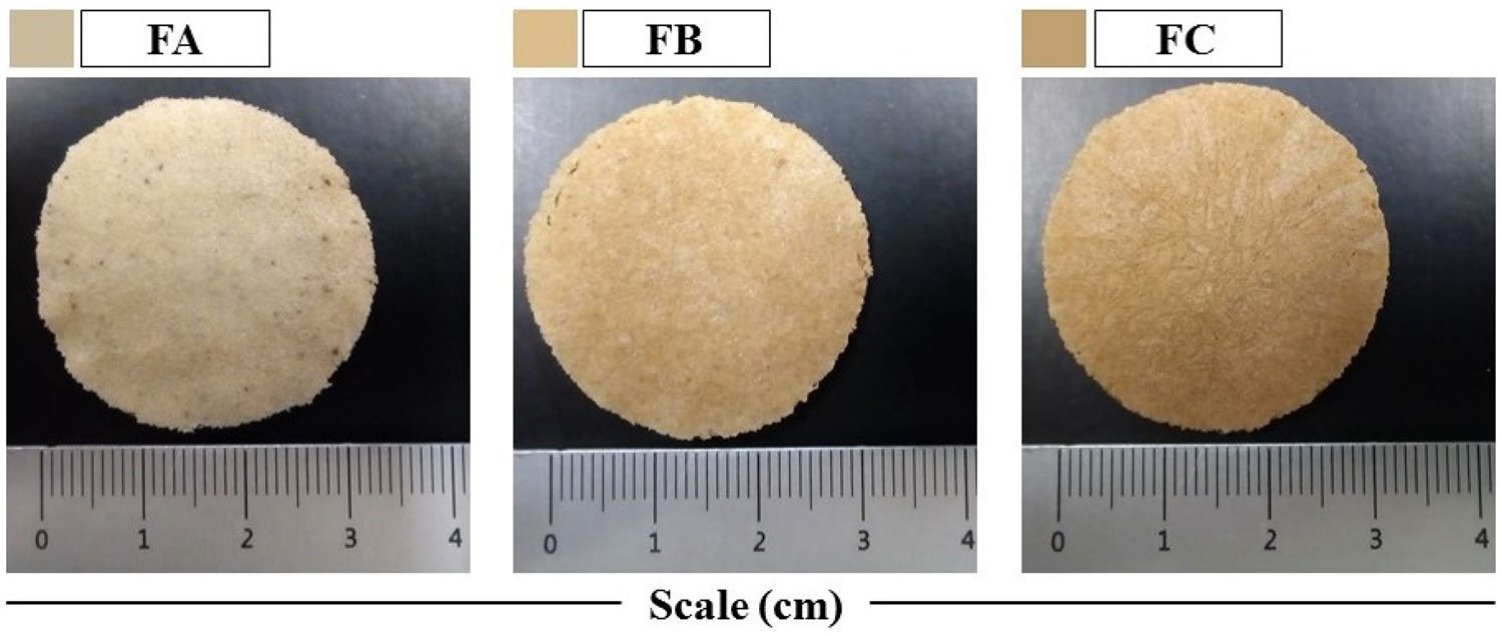
Macroscopic aspects of dense lamellar scaffolds. FA: COL-SF-PEG400-TPC (0.1%); FB: COL-SF-PEG400-TPC (0.3%); and FC: COL-SF-PEG400-TPC (0.5%)

Figure 1 shows the FA, FB, and FC scaffolds obtained after lyophilization. All formulations suggest efficient macroscopic aspects, such as integrity and resistance to manipulation. With the addition of TPC variations, it was possible to observe an increase in the shade of the scaffolds.

The scaffolds were sized using a stainless-steel caliper (LEE TOOLS-682626. São Paulo, Brazil) measuring 0.02 mm. The formulations had a thickness of 0.27 ± 0.03 cm, a radius of 1.72, and an area equivalent to ~ 9.3 cm^2^.

It must be noted that collagen has proven to be an effective scaffold for tissue engineering due to its high biocompatibility, low antigenicity, and biodegradability. However, one potential issue with using collagen gels for tissue engineering is their significant contraction when mixed with other biopolymers. Additionally, collagen gels are mechanically weak and prone to deformation [29]. Using plastic compression techniques combined with chemical crosslinking, collagen scaffolds have demonstrated improved mechanical stability, reduced swelling rate, and longer disintegration time [23].

Besides, a technique called compressed collagen gel has been developed for tissue engineering. Collagen gel is a biphasic material assembled up of a loose lattice structure filled with mostly fluid. The excess fluid can be removed from the gel by compressing it, which only takes a few minutes without affecting its biocompatibility and mechanical properties. This process creates a denser, stronger scaffold that can be easily shaped to meet specific requirements [30].

### Fourier transform infrared spectroscopy analysis

**Fig. 2:**
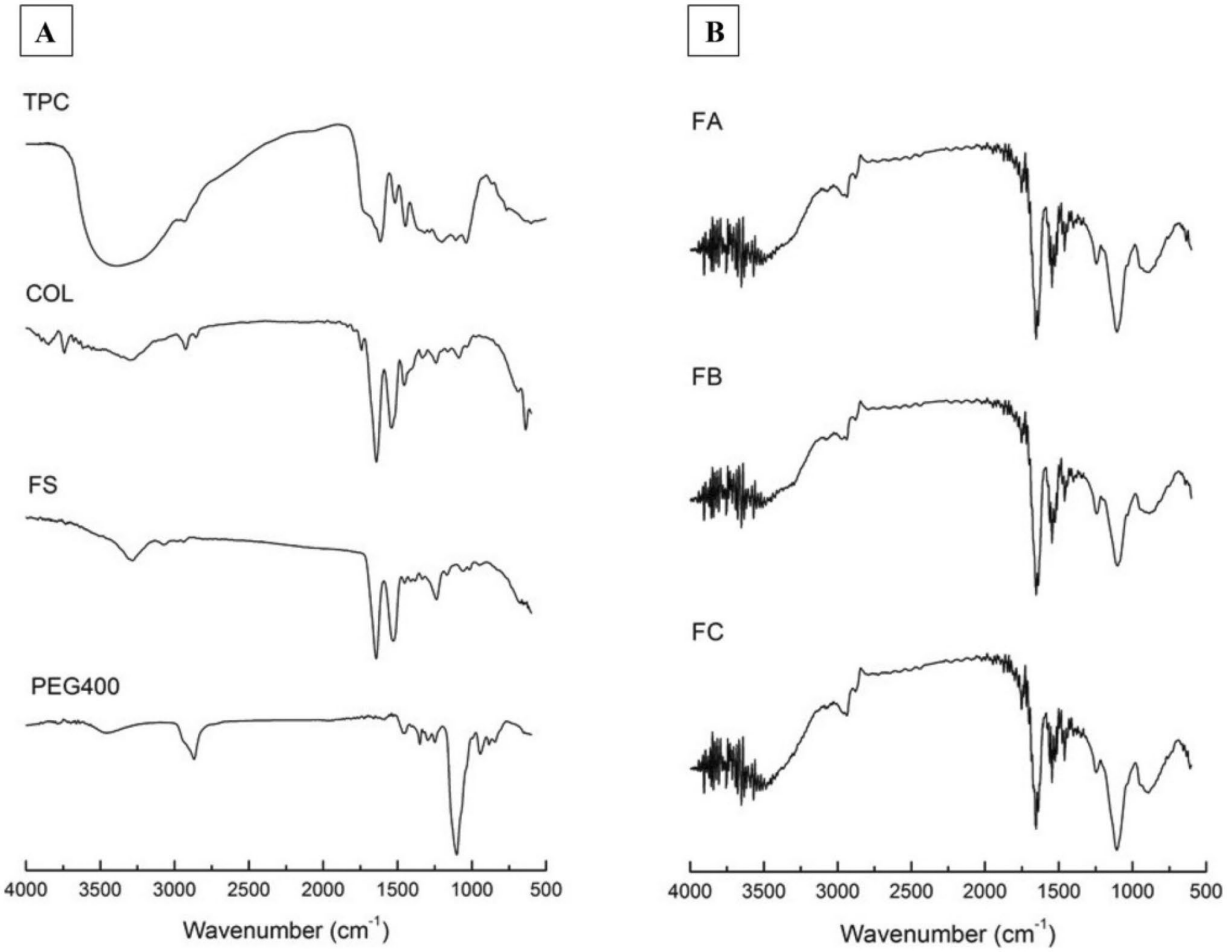
FTIR spectra of components (**A**) and dense lamellar scaffolds (**B**). Collagen (COL); silk fibroin (FS); polyethylene glycol 400 (PEG400), and total phenolic compounds (TPC). FA: COL-SF-PEG400-TPC (0.1%); FB: COL-SF-PEG400-TPC (0.3%), and FC: COL-SF-PEG400-TPC (0.5%)

The analysis of the infrared spectra of the components and scaffolds is shown in Fig. 2. FTIR is a technique that allows identifying organic and mineral substances through the functional groups present in the molecules and characterizing and indicating possible chemical interactions between substances (mixtures) [31].

In the spectrum of the guava leaf extract, the characteristic peaks of phenolic compounds (TPC) were observed in the regions at 3383 cm^−1^ assigned to the OH group and at 2931 cm^−1^, the asymmetric vibration of the aliphatic elongation structures. In the regions at 1616 cm^−1^ and 1517 cm^−1^, the CH and C = C elongation bands, typical of aromatic molecules, are assigned, respectively. The peak at 1446 cm^−1^ corresponds to antisymmetric bending in the CH_3_ plane. The peaks in the regions at 1201 cm^−1^, 1109 cm^−1^, and 1039 cm^−1^ correspond to the C–O stretching vibrations. Finally, CH’s peak at 765 cm^−1^ was attributed to out-of-plane conformations [26, 32, 33].

COL is the most present protein in the human body, basically composed of the amino acids glycine, proline, and hydroxyproline. The transmittance peaks characteristic of COL were observed in the regions at 1242 cm^−1^, typical of amide III, referring to C–N elongation and N–H deformation. The peak at 1537 cm^−1^, typical of amide II, attributed to vibrations in the bond plane N–H and C–N stretching, and the peak at 1648 cm^−1^ is typical of amide I, referring to the carbonyl elongation C = O. Finally, the peak in the region at 3302 cm^−1^ refers to the hydroxyl group (OH) [27, 34].

The SF spectrum showed characteristic peaks in the regions at 1238 cm^−1^ referring to amide III, associated with the N–C stretch, and at 1527 cm^−1^ denoting amide II, originating from the N–H deformation, structural conformation characteristic of silk I [35]. The peak at 1643 cm^−1^ refers to amide I, with the elongation of the carbonyl group (C = O) and amide II (1527 cm^−1^), corresponding to the structural conformation of silk II. Finally, the region at 3284 cm^−1^ refers to the OH group [27, 36].

The characteristic peaks of PEG 400 were observed in the regions of 1103 cm^−1^ representing the C–O–C group, 2868 cm^−1^ attributed to the elongation of the CH_2_ group, and 3452 cm^−1^ referring to the OH group [37].

In the spectra presented for the scaffolds manufactured by mixing COL, SF, PEG 400, and TPC polymers, it was possible to observe the persistence of some characteristic peaks of each component. However, the peaks were recorded with slight displacements and some overlaps. Regarding the COL and SF, it was possible to observe the prevalence of the amide groups (I, II, and III), but there was an overlap. Of the characteristic peaks of PEG 400, the prevalence of groups C–O–C and CH_2_ was observed. Concerning the TPC, the predominance of characteristic peaks was not observed. According to the results presented by the spectra, chemical interactions between the components of the scaffolds were confirmed.

The alterations observed in the transmittance spectra, mainly due to the absence of OH functional groups and the evidence of displacement and reduction of the intensity of the amide groups in the scaffolds, indicate that the groups are involved in the cross-linking of COL, occurring through the domain of hydrogen bonds to form the Schiff bases [23]. According to Radhakrishnan et al. [38], the amine and carboxylic functional groups are mainly responsible for the cross-linking reaction of COL.

In addition to the cross-linking of COL, it is also possible that cross-linking with the SF structure occurs. However, due to the application of a natural cross-linker, the reaction process is time-consuming, occurring mainly through lysine and arginine present in the protein structure. These amino acids represent a low percentage in the total composition of SF, about 0.6% in moles for each one [39, 40].

### Determination of the zeta potential of total phenolic compounds (TPC)

**Fig. 3:**
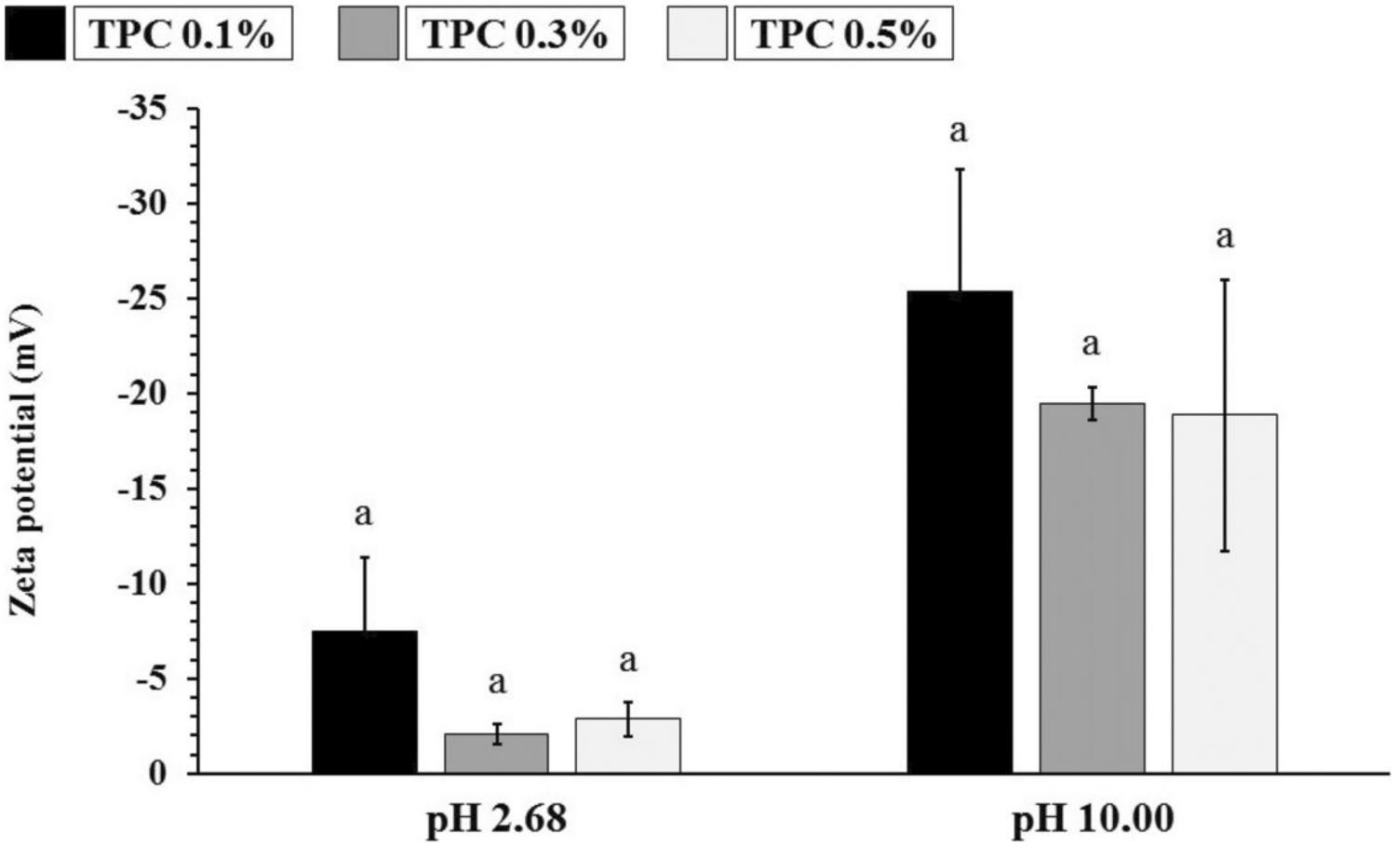
Zeta potential measurements of total phenolic compounds (TPC) solutions at pH 2.68 and 10.00

The zeta potential analyses of the aqueous solutions of TPCs were obtained in two moments, at the extraction pH (2.68) and the pH for the preparation of polymeric blends (10.00), as shown in Fig. 3. The zeta potential is a measure that allows determining the particles’ surface charge and indicates the thermodynamic stability of dispersed systems [41].

The values of the surface charge of TPCs in an aqueous solution were obtained, in general, showed a negative zeta potential. As we can see in the graph, the zeta potential remained negative regardless of the pH range. At extraction pH (2.68), the surface charges showed less negative zeta potential values: − 7.51 ± 3.88 mV (TPC 0.1%), − 2.06 ± 0.56 mV (TPC 0 0.3%), and − 2.87 ± 0.88 mV (TPC 0.5%); after correction for the reactional pH (10.00), the values became more negative − 25.34 ± 6.42 mV (TPC 0.1%), − 19.46 ± 0.85 mV (TPC 0.3%), and − 18.86 ± 7.14 mV (TPC 0.5%).

Provided that the composition in polyphenols present in the *Psidium guajava* L. is already documented in the scientific literature [42], the surface charge values presented by the TPCs are yet fundamental since the molecular structure of COL and SF consists of the junction of amino acids, with the prevalence of acid (COOH) and base (NH_2_) terminal and side groups, which gives these proteins the amphoteric characteristic. In this way, factors such as the pH of the reaction medium, temperature, and ionic strength will influence the structure of the molecule and may present prevalences with negative (COO^−^) or positive (NH3^+^) charge [43, 44].

**Fig. 4:**
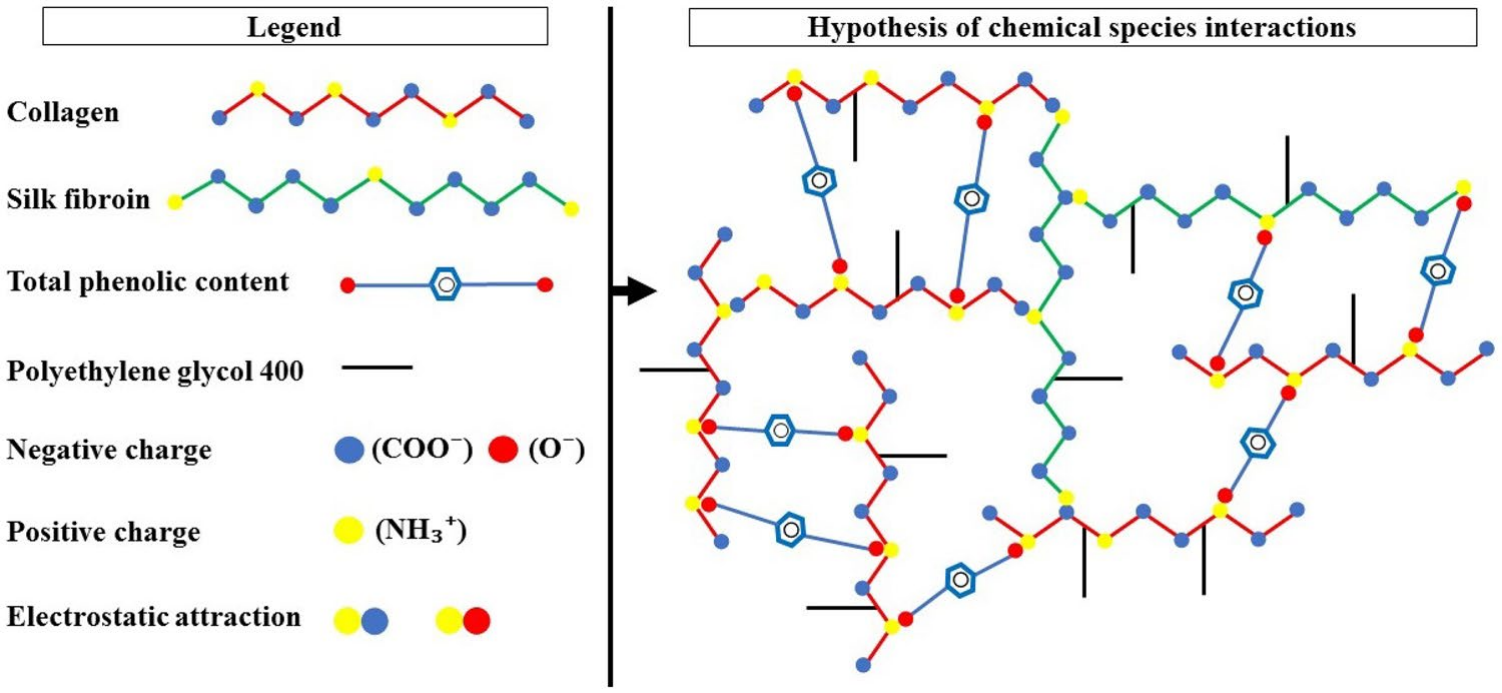
Interactions between chemical species of dense lamellar scaffolds hypothesis

Figure 4 presents the hypothesis of chemical interactions between the components of dense lamellar scaffold formulations using TPCs as a cross-linking agent.

The changes observed in the molecular representations of COL and SF in Fig. 4 were based on the isoelectric point of these components, and the isoelectric point of SF presented at pH between 3.6 and 5.1 [45], whereas the point isoelectric of COL is presented at pH between 7 and 8 [46]. With the correction of the reaction pH to 10, above the isoelectric point of proteins, negative charges are prevalent in the structure of amino acids. In this way, interactions by electrostatic attraction can occur between the carboxylic groups (COO^−^) and the amino groups (NH3^+^) existing in COL and SF, in addition to the cross-linking exerted by the TPCs (O^−^) with the amino groups (NH3^+^) existing in both proteins. The PEG 400 molecules in the formulation act as a plasticizing agent between the COL and SF chains, which are not interconnected by strong covalent primary forces, allowing them to slide freely one over the other [47].

The FTIR spectra and the values measured in the zeta potential corroborate the understanding of the chemical interactions between the components in the scaffolds formulation, mainly in the role of TPCs as a potential protein cross-linking agent.

### Differential scanning calorimetry

**Fig. 5:**
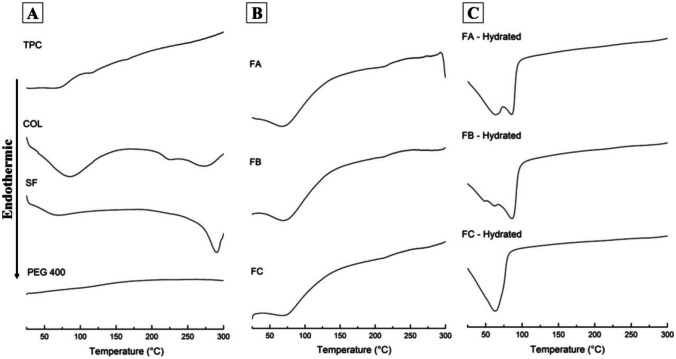
DSC thermoanalytical curves of components (**A**), dense lamellar scaffolds (**B**), and dense lamellar scaffolds hydrated (**C**). Collagen (COL); silk fibroin (SF); polyethylene glycol 400 (PEG400), and total phenolic compounds (TPC). FA: COL-SF-PEG400-TPC (0.1%); FB: COL-SF-PEG400-TPC (0.3%), and FC: COL-SF-PEG400-TPC (0.5%)

DSC is a thermodynamic tool for the direct assessment of absorbed (endothermic) or released (exothermic) thermal energy, in which the energy difference (heat flow) between a sample and a reference material (white) is measured as a function of the increase or decrease in temperature employing a differential calorimeter. The calorimetric analysis is mainly applied to monitor changes in the transition phase of pure substances or mixtures [48]. The thermoanalytical DSC curves of the raw materials used and the scaffolds formulations are shown in Fig. 5.

The calorimetric analysis performed for the COL showed three distinct thermal events, as previously reported by us [25]. The first thermal event was recorded at 85.74 °C with an onset of 39.75 °C and an end set of 124.75 °C, in addition to an ∆*H* =  − 203.80 J/g. This event is related to the loss of water absorbed in the protein fibers. The increase in temperature promotes the breakdown of cross-links that stabilize the native structure, causing its denaturation [49]. The second thermal event presented was attributed to a glass transition (*T*_*g*_) characterized by the displacement of the baseline, with heat capacity (*C*_*P*_) equivalent to 226.85 °C (onset: 217.41 °C; end set: 231, 98 °C) [27]. Finally, the third thermal event was observed at 272.64 °C with an onset of 253.73 °C, an end set of 300 °C, and ∆*H* =  − 27.02 J/g, suggesting a thermal decomposition reaction of the protein.

In the thermoanalytical curve of the SF, it was possible to observe two distinct thermal events. The first was recorded at 69.08 °C with an onset of 44.40 °C, an end set of 88.90 °C, and ∆*H* =  − 58.36 J/g. This thermal event was attributed to water evaporation, while the second was attributed to a thermal decomposition reaction of the material, with a thermal event recorded at 290.89 °C with an onset of 273.18 °C, and end set of 300 °C, and ∆*H* =  − 154.84 J/g [50].

The PEG 400 was impossible to record thermal events in the evaluated temperature range (25–300 °C). However, a study by Bertasi et al. [51] recorded a thermal event for PEG 400 at about 5 °C, which is related to the polymer’s melting point. In addition, there was no record of other events with increasing temperature (150 °C).

With this, it was possible to measure the denaturation temperature (*T*_*d*_) and the enthalpy of the scaffolds containing percentages of phenolic compounds, and the measurement of *T*_*d*_ corresponds to the cross-link density that occurred in each formulation. Figure 5B shows the scaffolds’ analytical curves, and the DSC’s numerical results are presented in Table 2.

**Table 2:** Temperature denaturation (*T*_*d*_) and enthalpies (∆*H*) of dense lamellar scaffolds (FA, FB, and FC)

	***T***_***d***_*** (°C)***	**Onset (°C)**	**End set (°C)**	**∆*H* (J/g)**
FA	67.28	46.08	109.88	− 187.65
FB	69.13	40.89	115.49	− 166.07
FC	67.62	39.63	110.21	− 155.02

FA: COL-SF-PEG400-TPC (0.1%); FB: COL-SF-PEG400-TPC (0.3%), and FC: COL-SF-PEG400-TPC (0.5%)

In the thermoanalytical curves of the dense lamellar scaffolds, similar *T*_d_ values were recorded in the FA and FC formulations, with the thermal event at 67.28 °C and 67.62 °C, respectively. However, an enthalpy variation of − 32.63 J/g was evidenced between the formulations (*FA* > *FC*). In the FB formulation, a *T*_*d*_ of 69.13 °C was recorded, showing a slight increase in temperature (1.68 ± 0.24 °C), corresponding to the cross-linking density. Compared with FA and FC, an enthalpy of − 166.07 was observed for FB, and this value is intermediate to the others (*FA* > *FB* > *FC*).

With respect to the values presented by COL alone, a reduction in *T*_*d*_ and enthalpy was observed in all formulations, regardless of the percentage of total phenolic compounds applied to the processing of each scaffold. This result states that incorporating the components caused changes in the values presented by the COL, reducing the *T*_*d*_ to 17.73 ± 0.98 °C and the enthalpy to 34.22 ± 16.60 J/g.

The calorimetric analysis of the scaffolds also showed a significant reduction in *T*_*g*_ at 226 °C, an event associated with the transition from the protein helix to the bobbin, indicating the extent of cross-linking [27]. This event occurs because the triple helix present in the COL structure is maintained by hydrogen bonds, and the cross-linking process stabilizes the helical structure, allowing the formation of intramolecular and intermolecular networks [52, 53]. Due to the interactions between the components, it was also possible to observe changes in the thermal decomposition reaction of the materials.

Thus, the temperature changes of *T*_*d*_, *C*_*P*_ enthalpy of *T*_*g*_ (226 °C), and decomposition reaction reaffirm the FTIR results, indicating the formation of a new structure rearrangement of the COL with the SF. The cross-linking action promoted by the total phenolic compounds and the PEG 400 molecules with plasticizing action, even in forming an immiscible blend between the polymers, controls the electrostatic attraction by PEG 400 in the sliding of the chains favors the balance of the system.

The samples were hydrated and characterized by thermal DSC assay to better understand the physicochemical behavior of the scaffolds for in vivo application (Fig. 5C). It was possible to observe significant changes in the peaks of the hydrated samples compared to the scaffolds’ thermoanalytical curves before and after hydration, where FA-hydrated presented a junction of two thermal events between 31.98 °C (onset) and 91.95 °C (end set), the first peak recorded at 63.58 °C and the second at 85.55 °C. In FB, a junction of three thermal events was observed between 29.76 °C (onset) and 93.93 °C (end set), the first peak recorded at 49.13 °C, the second at 62.39 °C, and the third at 86.11 °C. In FC, only one well-defined thermal event was observed, with a peak at 62.27 °C (onset: 29.98 °C; end set: 79.61 °C). Furthermore, the thermal event of *T*_*g*_ (226 °C) was not observed in any of the hydrated samples, which confirms the reduction in *T*_*g*_ and *C*_*P*_ due to increased water activity in the scaffolds.

In the thermograms shown by the scaffolds obtained with the addition of 0.1, 0.3, and 0.5% of TPC, average *T*_*d*_ values of 68.01 ± 0.98 °C were recorded (Table 2). However, after hydration, the scaffolds reduced *T*_*d*_ values of 5.26 ± 1.52 °C.

This result demonstrates the influence of the water activity contained in the hydrated scaffolds, making a counterpoint to the results evidenced in the scaffolds without the presence of TPC (Fig. 5, Table 2), where an increase of 14.00 ± 0.81 °C was observed in *T*_*d*_, indicating the need for more energy to remove the adsorbed water. Thus, the incorporation of TPC reduced the water activity. In addition, it suggested interactions with the phenolic structures present in the scaffolds, which underlies the formation of the other thermal events observed in the FA and FB samples.

### Scanning electron microscopy

**Fig. 6:**
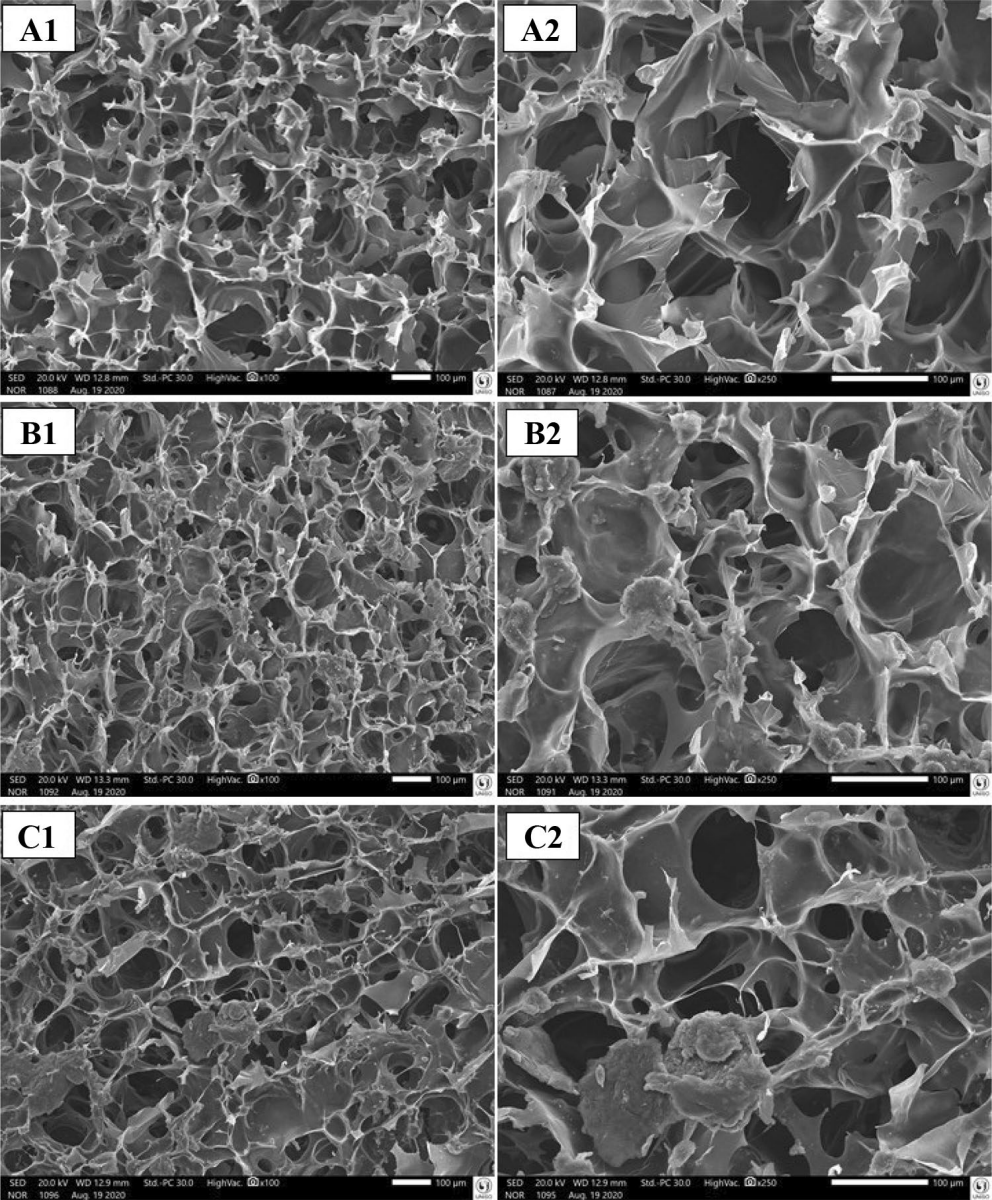
SEM of the FA (A1, A2), FB (B1, B2), and FC (C1, C2) dense lamellar scaffolds. FA: COL-SF-PEG400-TPC (0.1%); FB: COL-SF-PEG400-TPC (0.3%); and FC: COL-SF-PEG400-TPC (0.5%)

The morphological characteristics of the dense lamellar scaffolds FA, FB, and FD were investigated by SEM and are shown in Fig. 6. The technique based on a temperature gradient involves freezing a water-based polymeric solution or suspension and removing ice crystals formed through sublimation (lyophilization) [54]. In our study, the morphology of the upper face of the scaffolds showed highly porous microstructures at all magnifications evaluated.

The pore diameter of the scaffolds estimated from the SEM micrographs showed a size distribution between 46.19 and 129.70 µm in the FA formulation (Fig. 6A1, A2). For the FB formulation (Fig. 6B1, B2), pore size distributions between 31.56 and 84.53 µm were presented, while in the FC formulation (Fig. 6C1, C2), pore sizes between 41.54 and 106.00 µm were presented.

According to Griffon et al. (2006), the pore size suitable for the growth of chondrocytes applied in cartilage tissue repair is between 70 and 120 µm. In a study, by Stenhamre et al. (2011), scaffolds with different pore sizes, < 150 µm, 150–300 µm, and 300–500 µm were developed and seeded with chondrocytes and subjected to in vitro chondrogenic induction. During cell culture, no empty pores were seen in the scaffold with a pore diameter < 150 µm. Chondrocytes filled the pores and produced an extensive extracellular matrix.

In another study, Nava et al. [55] prepared scaffolds with three different geometries, the first with a pore diameter ranging between 50 and 100 µm (average = 75 µm), the second with a pore diameter between 100 and 150 µm (average = 125 µm), and the third between 150 and 200 µm (mean = 175 µm). The results show that the combination of smaller scaffold pores is more effective in promoting chondrogenesis.

These results confirm that the pore size obtained in the structure of the dense lamellar scaffolds developed in this study is viable for applications in cartilage tissue engineering.

Another critical point observed in the micrographs of the scaffolds is related to the 3D structure. It is suggested that the cross-linking process exerted by the incorporation of total phenolic compounds associated with the plastic compression technique and the temperature gradient applied during the nucleation phase and subsequent sublimation by lyophilization resulted in the formation of a 3D structure with a gyroid-like geometry.

The gyroid geometry was discovered in 1970 by Alan Schoen. It is described as a surface topology that meets the minimum triple periodic surface requirements that do not intersect or contain straight lines [56]. In a study developed by Olivares et al. [57], two distinct morphologies of scaffolds were compared, gyroid and hexagonal structures. According to the research results, obtaining a gyroid structure with better cell seeding and nutrient transport is more probable than in hexagonal structures. However, gyroid structures induce less homogeneous mechanical stimuli on the scaffold surface than hexagonal structures.

Following the structure relationship and cell contact, Downing et al. [58] point out that gyroid has been identified as a suitable cell topology for engineering applications, particularly in its solid lattice form, for biomedical applications, offering additional benefits of a continuous surface that divides space and provides ample surface area for transfer of energy heat or cell fixation.

The characterizations obtained by SEM collaborate with the relationship between surface structure and pore size. The µCT assay can verify the parameters of porosity, interconnectivity, pore volume, and anisotropic degree. These morphological characteristics of scaffolds are a crucial factor, directly influencing the behavior of cells and helping in cell adhesion, proliferation, differentiation, and orientation [59, 60].

### Computed microtomography (µCT)

**Fig. 7:**
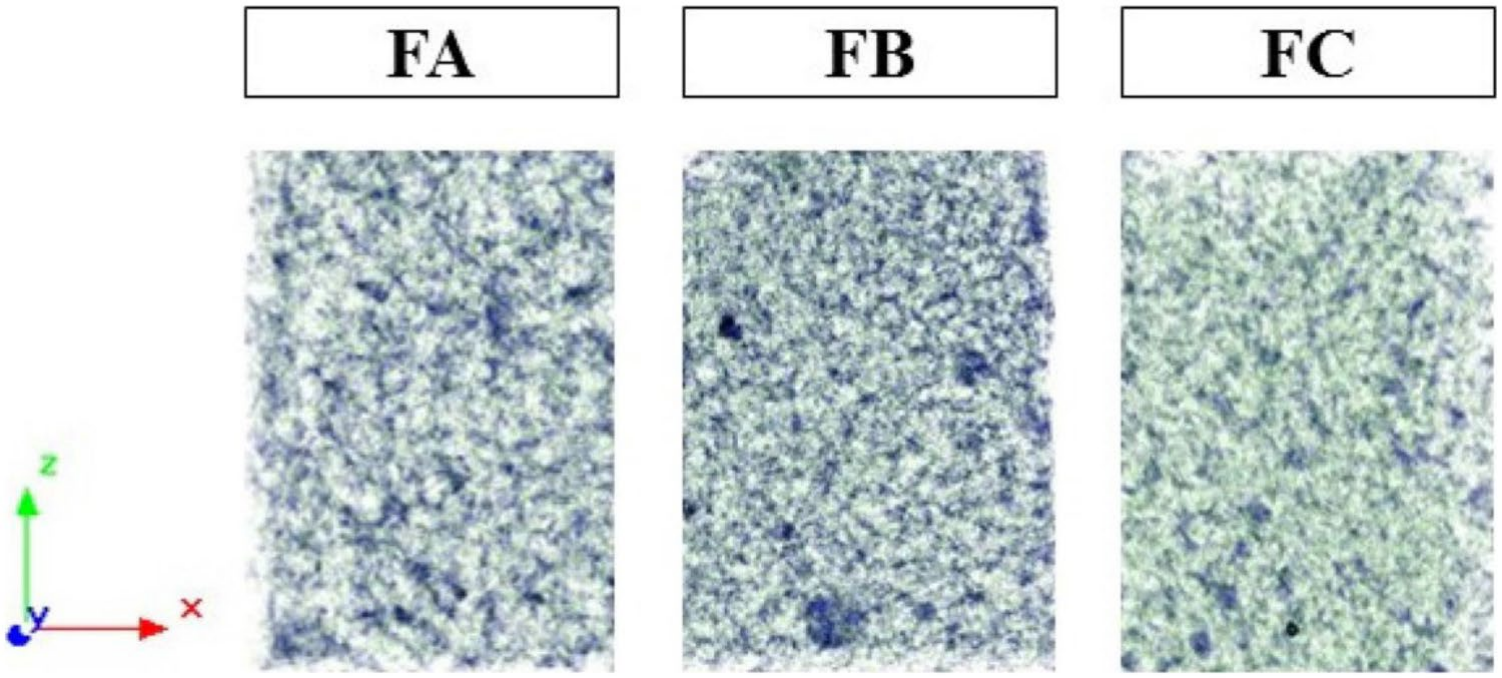
Morphological characteristics of dense lamellar scaffolds evaluated by µCT. FA: COL-SF-PEG400-TPC (0.1%); FB: COL-SF-PEG400-TPC (0.3%); and FC: COL-SF-PEG400-TPC (0.5%)

The 3D morphometric characteristics of the dense lamellar scaffolds FA, FB, and FC were obtained by µCT and are shown in Fig. 7. The morphometric results corroborate the morphological characteristics evidenced by SEM (Fig. 6), confirming through the density contrast of the scaffolds the singularities of each porous structure. In addition to these characteristics, the µCT assay can measure values related to the 3D structure of the scaffolds, such as interconnectivity, the volume of open pores, the percentage of closed pores, and the anisotropic degree (Table 3). Table 3 presents the morphological characteristics of the dense lamellar scaffolds. In addition to the porous surface structure, the scaffolds formed a matrix with the interconnectivity of the pores, confirming a higher percentage for the FA formulation (74.92%).

**Table 3:** Morphometric data of dense lamellar scaffolds

	**FA**	**FB**	**FC**
Total VOI volume (mm^−1^)	8.42	8.54	15.33
Pore interconnectivity (%)	74.92	72.64	72.70
Volume of open pores (mm^−3^)	6.31	6.20	11.14
Open porosity (%)	74.90	72.61	72.67
Closed porosity (%)	0.09	0.09	0.10
Degree of anisotropy	0.97	0.84	0.09

FA: COL-SF-PEG400-TPC (0.1%); FB: COL-SF-PEG400-TPC (0.3%); and FC: COL-SF-PEG400-TPC (0.5%)

The analysis also made it possible to measure the volume of open pores and the percentage of closed pores. Incorporating TPC in the formulations did not result in significant variations in the percentage of closed pores (0.09–0.10%). However, when compared to the volume of open pores, a greater volume (11.14 mm^−3^) was observed for the FC formulation, obtained by incorporating 0.5% of TPC in the polymeric mixture. These parameters play a crucial role, as cells depend on the interconnectivity and volume of open pores for nutrient permeation and oxygenation, as well as for orientation and, consequently, proliferation throughout the scaffold structure [61].

Finally, the anisotropic degree was evaluated since it indicates how the formation of new tissue in the scaffolds structure will be oriented. The degree is measured between 0 and 1, where 0 corresponds to a fully isotropic structure, while 1 corresponds to a completely anisotropic structure [62]. The organization presented by ECM fibers within fabrics can vary from anisotropic (fiber alignment, directional amplification of binder density) to isotropic (randomly aligned, uniform binder availability) [63].

The values expressed by the anisotropic degree of the dense lamellar scaffolds showed that the incorporation of TPC in the polymeric mixtures COL-SF-PEG 400 with percentages of 0.1% (FA) and 0.3% (FB) resulted in anisotropic structures with values of 0.97 (FA) and 0.84 (FB), with a slight reduction in the degree observed with the increase in the percentage of TPC.

When the percentage of 0.5% of TPC was incorporated, the scaffold structure showed a significant reduction in the anisotropic degree, resulting in an isotropic structure (0.09). Nano and microscale topographical features have been recognized as crucial cell growth, migration, and phenotype regulators. For tissue engineering, an utterly biomimetic architecture aims to mimic the high degree of spatial organization of the ECM and basement membrane components [64].

However, the components that form the tracheal structure, such as mucosa, submucosa, adventitia, cartilage, and smooth muscle, present considerable complexity. This complexity has attracted great scientific interest in elucidating some aspects of the material's behavior (isotropic vs. anisotropic) [65].

According to a study by Teng et al. [66], the mucous and submucosal layer trachea structures present stretching and stretching with anisotropic behavior. However, other structures of the trachea showed opposite characteristics. According to Safshekan et al. [65], the three main components of the trachea, such as cartilage, smooth muscle, and connective tissue, showed isotropic behavior.

Therefore, the characteristics presented by the dense lamellar scaffolds with percentages of TPC indicate a potential for both recovery of isotropic structures (FC) and anisotropic structures (FA and FB). However, aiming at the application in tracheal tissue engineering for tissue recovery due to surgical tumor removal, the isotropic behavior presented by FC is more indicated.

### Mechanical properties

**Fig. 8:**
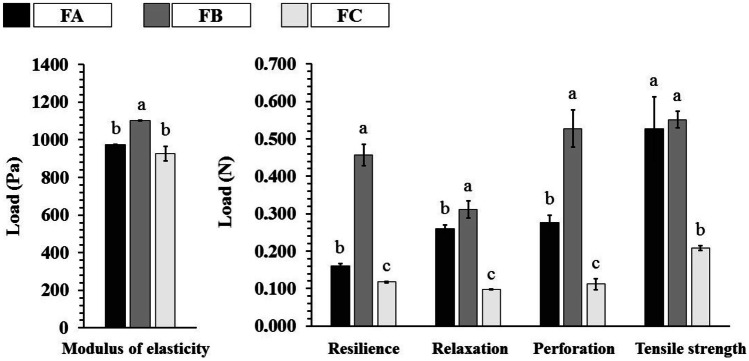
Mechanical properties of dense lamellar scaffolds. FA: COL-SF-PEG400-TPC (0.1%); FB: COL-SF-PEG400-TPC (0.3%); and FC: COL-SF-PEG400-TPC (0.5%). Equal letters (for the same analysis) indicate that there is no significant difference between the mean values (*p* > 0.05; *n* = 3)

The mechanical properties represent an essential set of characteristics for the successful application of a scaffold. The scaffold structure needs to withstand the stresses exerted during the surgical implantation of the device and maintain the necessary microenvironment for cell growth [67, 68]. Figure 8 shows the results of the mechanical properties of the FA, FB, and FC scaffolds for the elastic modulus (Young’s modulus), resilience, relaxation, perforation, and tensile strength tests.

The elastic modulus or Young’s modulus refers to the mechanical property that measures the stiffness of a given material. Young’s modulus is obtained by the ratio between the stress applied to the material and the deformation associated with the applied stress [69]. The analysis of the results showed that the scaffolds FA (974.09 ± 79.00 Pa) and FC (927.97 ± 38.66 Pa) presented statistically similar values (*p* > 0.05), among the three formulations, the FB showed greater stiffness (1103.29 ± 38.04 Pa).

The resilience, relaxation, perforation, and tensile strength analyses (Fig. 8) showed changes in both scaffolds due to the addition of TPC percentages, resulting in higher nominal values about the formulations *FB* > *FA* > *FC* and through statistical analysis. The FC presented a statistically significant difference (*p* < 0.05) in these trials. The only similarity between the FA (0.527 ± 0.086 N) and FB (0.551 ± 0.021 N) scaffolds in the tensile strength test was found.

These results demonstrate the influence of the orientation of the porous structure (anisotropic degree) on the scaffolds, confirming significant differences for the FC formulation (isotropic) in the mechanical properties, except for the elastic modulus.

For the scaffold to be successful in tissue recovery, several factors must be considered, such as surface morphology, pore size, mechanical properties versus porosity, and adequate biodisintegration. However, of these factors, the importance of mechanical properties in cell growth is undeniable in tissue engineering applications such as bones, cartilage, blood vessels, tendons, and muscles [70].

Tracheal cancer, even a rare disease, has a curative therapeutic option when the tumor is localized [7]. Thus, complete surgical resection of the tumor represents the best treatment option, with greater results in terms of overall survival [8].

Therefore, it is interesting from an application point of view that the mechanical properties of dense lamellar scaffolds can perform a physical function similar to the trachea structure and, thus, guide the recovery of native tissue after tumor removal.

### Swelling efficiency

**Fig. 9:**
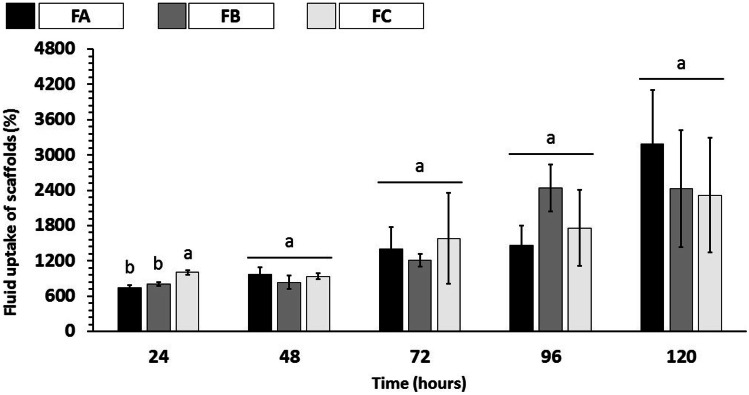
Fluid absorption from dense lamellar scaffolds (%), fluid retained throughout the scaffold structure in PBS (pH 7.4 at 37 °C). FA: COL-SF-PEG400-TPC (0.1%); FB: COL-SF-PEG400-TPC (0.3%); and FC: COL-SF-PEG400-TPC (0.5%). Equal letters (for the same analysis) indicate that there is no significant difference between the mean values (*p* > 0.05; *n* = 3)

**Fig. 10:**
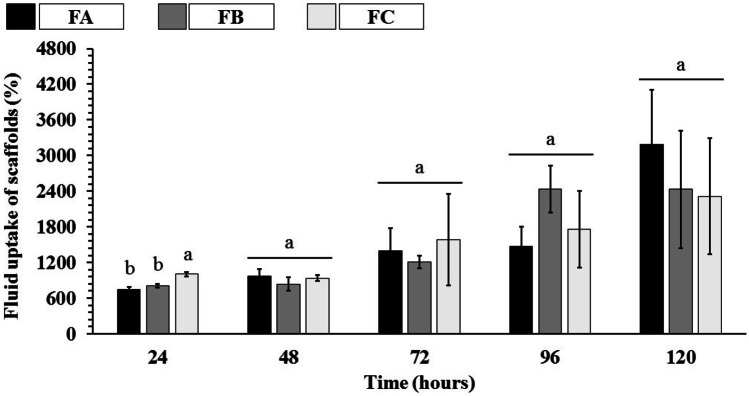
Fluid absorption of dense lamellar scaffolds (%) by the scaffold material in PBS (pH 7.4 at 37 °C). FA: COL-SF-PEG400-TPC (0.1%); FB: COL-SF-PEG400-TPC (0.3%); and FC: COL-SF-PEG400-TPC (0.5%). Equal letters (for the same analysis) indicate that there is no significant difference between the mean values (*p* > 0.05; *n* = 3)

The potential applications in tissue engineering, the swelling profile is a crucial parameter to evaluate the behavior of scaffolds properties. When applied, these devices will be in intimate contact with blood and other body fluids. In this way, an adequate swelling profile can allow, in addition to the absorption of body fluids, the transfer of signaling molecules, oxygenation, and nutrients to the scaffold matrix [71, 72]. In this way, the swelling profile of the scaffolds was evaluated in a buffer solution (PBS pH 7.4 at 37 °C). Figure 9 shows the swelling characteristics related to the fluid absorbed and retained by the entire scaffold structure (matrix + pores), while Fig. 10 shows the swelling characteristics by the scaffold material itself (matrix).

The analysis of the results of total swelling of the scaffold (matrix + pores) showed that in 24 h of immersion in PBS, the FC formulation presented a higher percentage of swelling (1005.41 ± 36.57%), while the FA formulations (742.87 ± 40.10%) and FB (802.47 ± 32.33%), despite the different nominal values, did not present statistically significant differences (*p* < 0.05). During the study periods, it was possible to verify an increase in the swelling values of the scaffolds. However, the results recorded between 72 and 120 h did not show significant statistical differences (*p* < 0.05), regardless of the percentage of TPC used in the scaffolds.

Regarding the swelling of the scaffolds polymeric material (Fig. 10), a gradual increase in the swelling values was also observed. However, despite the different nominal values recorded in each study period (24–120 h), there was no statistically significant difference between formulations (*p* > 0.05).

The results obtained in the tests, both for the entire scaffold structure (matrix + pores) and for the scaffold material itself (matrix), collaborate to mimic the possible behavior in vivo. Despite the percentages of TPC applied in each formulation and the isotropic orientation confirmed in FC (Table 3), the swelling values recorded were statistically similar to FA and FC (anisotropic).

Measuring swelling is desirable in tissue engineering and biomedical applications, as fluid absorption can initially lead to increased pore size and porosity of scaffolds, reflecting the expansion of the internal contact area for infusion and cell fixation [73].

### In vitro biodisintegration study

**Fig. 11:**
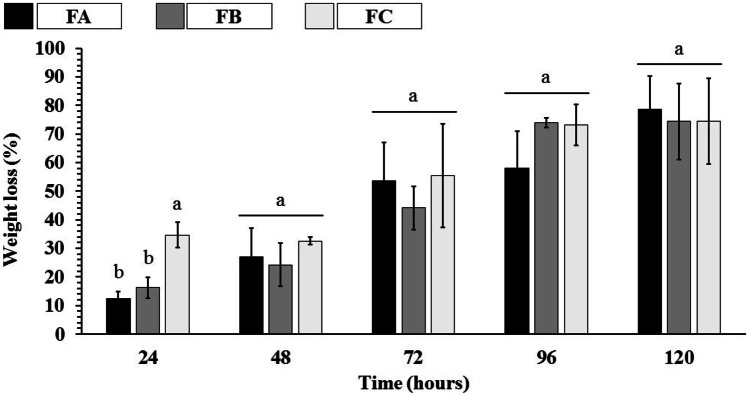
Weight loss (%) of dense lamellar scaffolds in PBS (pH 7.4 at 37 °C). FA: COL-SF-PEG400-TPC (0.1%); FB: COL-SF-PEG400-TPC (0.3%); and FC: COL-SF-PEG400-TPC (0.5%). Equal letters (for the same analysis) indicate that there is no significant difference between the mean values (*p* > 0.05; *n* = 3)

Figure 11 shows the scaffolds’ disintegration relationship as a time function. Measuring the rate of biodisintegration of scaffolds is crucial, as they directly influence the integrity of the device structure over time and the regeneration or remodeling performed by native cells in vivo. In this sense, a method widely applied to preliminarily simulate the rate of biodisintegration of scaffolds is incubation in buffer solution (PBS pH 7.4 at 37 °C).

The analysis of the results showed that the weight loss rate (%) of the FA and FB formulations was similar throughout the study (*p* > 0.05). The FC formulation, on the other hand, presented the highest value of weight loss in 24 h (*p* < 0.05); however, after this period, no statistically significant differences were observed (*p* > 0.05) when compared to the other scaffolds.

As observed in the evaluation of the swelling profile of the scaffolds (Figs. 9 and 10), the percentages of TPC applied, as well as the orientation of the structure (isotropic or anisotropic), did not influence the disintegration rate of the formulations, presenting statistically similar values after the 48 h (*p* > 0.05). In this way, the results indicate that the biodisintegration occurred exclusively due to the scission of the long molecular chains of the polymers that compose it. This scission is caused by chemical reactions, mainly hydrolytic reactions, resulting in low molecular mass and loss of mass of the polymers [74]. Notably, the product(s) of the scaffolds’ biodisintegration must be non-toxic and easily absorbed or excreted via metabolic pathways [75], as happens with any other material aimed for intracellular delivery [76–78].

**Fig. 12:**
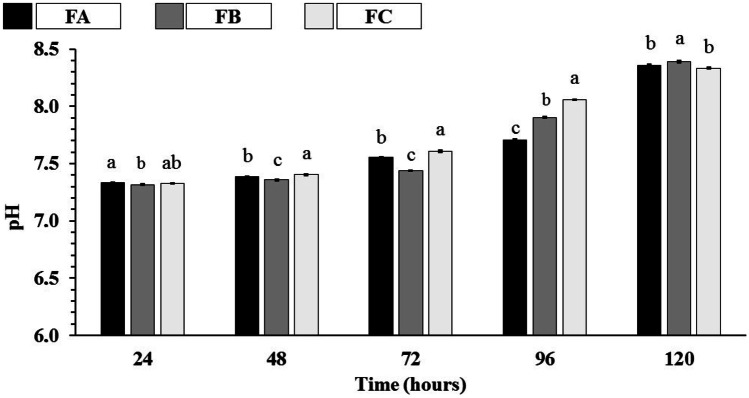
Dense lamellar scaffolds pH in PBS at 37 °C. FA: COL-SF-PEG400-TPC (0.1%); FB: COL-SF-PEG400-TPC (0.3%); and FC: COL-SF-PEG400-TPC (0.5%). Equal letters (for the same analysis) indicate that there is no significant difference between the mean values (*p* > 0.05; n = 3)

Figure 12 shows the pH values recorded during the in vitro biodisintegration study. During the study, the generation of biodisintegration products through hydrolytic reactions caused an imbalance in the pH of the PBS, recording significant increases in the pH scale after 72 h of the study.

The scaffolds formulations showed a pH range between 7.33–8.36 (FA), 7.32–8.39 (FB), and 7.33–8.33 (FC) over 120 h of the study evidencing a variation in the pH scale ≥ 1.00. Due, in particular, to the presence of total phenolic compounds in the scaffolds, as their chemical structures were disintegrating over time and generating fragments with base characteristics, resulting in the alkalization of the incubation medium.

Measuring the effect of the products generated during the biodisintegration process on pH is of great importance since acid–base balance is essential in the remodeling process in tissue regeneration processes [79]. According to Wu et al. [80], extracellular pH directly affects energy metabolism and ECM synthesis produced by chondrocytes. Thus, further investigations are needed to measure the behavior of chondrocytes in vitro under the conditions presented by the scaffolds since the increase in pH was verified gradually, besides the biocompatibility and cytotoxicity [76–78], with the proof of concept in an in vivo model of the disease.

## Conclusions

The plastic compression technique produced a dense lamellar scaffold based on collagen, silk, polyethylene glycol 400, and total phenolic compounds efficiently. The addition of TPC percentages modulated the scaffolds’ morphological characteristics, morphometric data, and mechanical properties. The FTIR spectra show chemical interactions between the polymers, and the application of TPC as a cross-linking agent, exerting ionic bonds between the amine and carboxylic functional groups of the COL. In addition, it was possible to cross-link the SF even in a smaller amount. The allowed conformational transition from the α-helix and random coil to the β-sheet structure is also allowed. The DSC analysis confirmed the occurrence of chemical interactions, promoting a new structural conformation. The images captured by µCT and SEM showed that the scaffolds have porosity, interconnectivity, and porous surface structure with gyroid-like geometry. However, the anisotropic degree was modulated according to the percentage of TPC applied, presenting anisotropic (FA and FB) and isotropic (FC) structures. The effect of TPC on swelling capacity and biodisintegration rate showed similarities between the formulations. In the mechanical properties, higher strengths were confirmed for FB (0.3% of TPC) and lower for FC (0.5% of TPC). Therefore, with the results obtained, we can confirm that the percentages of total phenolic compounds applied could modulate the properties of the scaffolds, mainly morphological, morphometric, and mechanical data, resulting in dense lamellar scaffolds with characteristics of interest for application in tracheal tissue engineering. Thus, this study may provide a way to improve the regeneration of the main tracheal structures (cartilage, smooth muscle, and connective tissue) after surgical tumor removal. We have successfully explored the possibility of replacing cytotoxic crosslinking agents commonly used in scaffold preparation with another biocompatible alternative, besides assessing if phenolic crosslinkers can provide the necessary structural features for scaffolds, such as mechanical and structural properties, proper porosity, and interconnectivity between pores to enable free fluid circulation.

## Data Availability

The datasets generated during and/or analyzed during the current study are available from the corresponding author on reasonable request.
